# *In Silico* and Fluorescence *In Situ* Hybridization Mapping Reveals Collinearity between the *Pennisetum squamulatum* Apomixis Carrier-Chromosome and Chromosome 2 of Sorghum and Foxtail Millet

**DOI:** 10.1371/journal.pone.0152411

**Published:** 2016-03-31

**Authors:** Sirjan Sapkota, Joann A. Conner, Wayne W. Hanna, Bindu Simon, Kevin Fengler, Stéphane Deschamps, Mark Cigan, Peggy Ozias-Akins

**Affiliations:** 1 Department of Horticulture, University of Georgia-Tifton Campus, Tifton, Georgia, 31793, United States of America; 2 Department of Crop and Soil Sciences, University of Georgia-Tifton Campus, Tifton, Georgia, 31793, United States of America; 3 DuPont Pioneer, DuPont Pioneer, Johnston, Iowa, 50131, United States of America; 4 DuPont Experimental Station, Wilmington, Delaware, 19803, United States of America; University of Perugia, ITALY

## Abstract

Apomixis, or clonal propagation through seed, is a trait identified within multiple species of the grass family (*Poaceae*). The genetic locus controlling apomixis in *Pennisetum squamulatum* (syn *Cenchrus squamulatus*) and *Cenchrus ciliaris* (syn *Pennisetum ciliare*, buffelgrass) is the apospory-specific genomic region (ASGR). Previously, the ASGR was shown to be highly conserved but inverted in marker order between *P*. *squamulatum* and *C*. *ciliaris* based on fluorescence *in situ* hybridization (FISH) and varied in both karyotype and position of the ASGR on the ASGR-carrier chromosome among other apomictic *Cenchrus/Pennisetum* species. Using *in silico* transcript mapping and verification of physical positions of some of the transcripts via FISH, we discovered that the ASGR-carrier chromosome from *P*. *squamulatum* is collinear with chromosome 2 of foxtail millet and sorghum outside of the ASGR. The *in silico* ordering of the ASGR-carrier chromosome markers, previously unmapped in *P*. *squamulatum*, allowed for the identification of a backcross line with structural changes to the *P*. *squamulatum* ASGR-carrier chromosome derived from gamma irradiated pollen.

## Introduction

Most current grass species are found to be derived from a common ancestor that lived about 50–80 million years ago. Despite the relatively recent and monophyletic origin of the grass genomes, there is considerable divergence in genome size and chromosome number [1, 2]. Yet sequenced members of the Poaceae clade have shown conservation of gene order (collinearity) among species such as rice, sorghum, maize, and foxtail millet [3]. Pearl millet (*Pennisetum glaucum*) shares a common ancestor with foxtail millet (*Setaria italica*) ~8.3 million years ago and with maize and sorghum ~26 million years ago [4].

There are several apomictic species in the genus *Cenchrus/Pennisetum*. Apomixis has been defined as asexual reproduction through seed [5]. Apomixis in *P*. *squamulatum* (syn *Cenchrus squamulatus*) and the closely related species *C*. *ciliaris* (syn *Pennisetum ciliare*) was found to be controlled by a dominant and hemizygous genetic locus named the apospory-specific genomic region (ASGR) [6, 7]. The ASGR in *P*. *squamulatum* is identified as a large (>50 Mb in size), heterochromatic chromosomal block localized near the telomere on the short arm of the ASGR-carrier chromosome by fluorescence *in situ* hybridization (FISH). The ASGR contains a region of low copy DNA flanked by regions of high copy DNA [8]. Physical mapping of the ASGR between *P*. *squamulatum* and *C*. *ciliaris* identified an inversion but conservation of ASGR-BAC order between the two species [9]. Comparison of the ASGR across other apomictic *Cenchrus/Pennisetum* species revealed variation in the ASGR chromosomal position and ASGR-carrier chromosome structure, suggesting movement and divergence of the ASGR block within the *Cenchrus/Pennisetum* lineage [10]. Within the ASGR, partial sequencing of ASGR-linked BAC clones showed the presence of multiple regions of small-scale, but not large-scale, collinearity with the rice and sorghum genomes [11].

An apomictic backcross (06-A-58) of *P*. *glaucum*, originating from a cross between *P*. *squamulatum* and tetraploid *P*. *glaucum*, was identified by FISH to carry one alien chromosome, the ASGR-carrier chromosome from *P*. *squamulatum* [12]. Forty-nine contigs, generated from the assembly of 454 sequences derived from dissected apomictic ovules, were mapped to the ASGR-carrier chromosome via SCAR, CAPS or SSCP markers [13]. Contig PS26_c9369 demonstrated tight linkage to the ASGR. Three other contigs (PS26_c5080, PS26_c33813, and PS26_c2552) mapped as unlinked to the ASGR using CAPS markers. The remaining contig SCAR markers failed to identify polymorphisms for mapping within a segregating F_1_ population. However, *in silico* mapping of the 49 ASGR-carrier chromosome contigs to the sorghum reference genome identified 21 sequences (BlastN, e value ≤e ^-20^) with similarity to the sorghum genome of which 17 had a unique or highest similarity to sorghum chromosome 2.

As the 454 transcriptome data was 3ʹ biased due to T7 amplification of the ovule RNA, we generated additional transcriptome assemblies using RNA-seq data from an apomictic backcross and screened these to extend the length of the 454 ASGR-carrier chromosome contigs for additional comparison to the sorghum and foxtail millet genomes. The predicted *in silico* positions of 7 contigs from the *P*. *squamulatum* ASGR-carrier chromosome, based on hits to the sorghum and foxtail millet genome, were verified by cytogenetic mapping of BAC clones containing the SCAR marker for the ASGR-carrier chromosome contigs. Our analysis demonstrates that the ASGR-carrier chromosome from *P*. *squamulatum*, outside the ASGR boundary, is collinear with chromosome 2 of sorghum and foxtail millet. Using the established ASGR-carrier chromosome SCAR markers and physical mapping results, a screen of gamma irradiated offspring was tested to identify lines with structural changes to the ASGR-carrier chromosome. This screen identified a sexual line which has lost the ASGR, but retained most of the long arm of the ASGR-carrier chromosome.

## Materials and Methods

### Plant material

Apomictic *P*. *glaucum* backcross 8 (BC_8_) (06-A-58) derived seedlings carrying the ASGR-carrier chromosome [12] were used for FISH and tissue collection for RNA extraction. Backcross 06-A-58 is a facultative apomict and therefore produces progeny derived through both modes of reproduction, apomictic and sexual. DNA from seedlings was extracted using a modified CTAB method [14] and screened for the ASGR using the ASGR-linked SCAR marker p787/788 [12].

### Extension of ASGR-carrier chromosome transcript information

RNA was extracted from unfertilized ovaries of apomictic and sexual BC_8_ (06-A-58) derived plants collected on the day of anthesis (anther exsertion). Approximately 50 ovaries were collected from individual plants based on their mode of reproduction. RNA was isolated from 12 apomictic and 12 sexual plants usingQiagen Plant RNAeasy kit. RNA from individuals was pooled based on mode of reproduction to make an apomictic and sexual RNA sample for sequencing. Sequencing libraries were constructed according to manufacturer’s instructions and Illumina sequenced to yield 2 x 76 paired-end reads. Sequences from both libraries were quality trimmed, separated into paired-end and single-end reads (~100 million reads for each library), and assembled together with the Velvet *de novo* [15] assembly algorithm. Two different assemblies were used (varying in K-mer value) to identify the longest ASGR-carrier chromosome transcript available. PS contig sequences used for *in silico* analysis are located in S1 File.

### Identification of ASGR-carrier chromosome BACs

BACs linked to the ASGR-carrier chromosome were identified by screening the polyhaploid BAC library [16] with probes derived from the ASGR-carrier chromosome transcripts [13]. Hybridizing BAC clones were confirmed as linked to the ASGR-carrier chromosome via PCR amplification with the respective ASGR-carrier chromosome SCAR marker. Markers with isolated BACs are indicated in Table 1.

**Table 1:** Information for *in silico* and deletion mapping line.

SCAR Primers^a^	PS26 contig	454 contig length (bp)	454 contig hit to sorghum genome	Velvet contig length (bp)	Sorghum hit	e-value	Sorghum start	FT Millet Hits	e-value	FT Millet start	Presence of SCAR marker in the 312 line
1538/1539	c17388	209	n	527	No hit			No hit			yes
1498/1499	c30691	219	n	615	No hit			No hit			N/A
1514/1515* (ASGR-linked)	c9369	330	y	515	chr-3, 4, 6, &10	~6.83E-35 to 9.88E-45		chr-1	3.00E-46	23,795,001	no
1476/1477	c10331	301	n	1635	chr-9 chr-2	4E-38 6E-35	59,306,986 71,178,293	chr-6	4.00E-113	5,498,118	no
1478/1479	c11544	237	n	544	chr-1	8.00E-75	30,659,990	chr-9	7.00E-92	20,435,700	no
1604/1605* (m/h)	c194	478	n	1425	No hit			chr-2	0	5,554,462	no
1567/1568	c1422	397	y	512	chr-2	4.00E-114	8,168,376	chr-2	0	7,323,838	N/A
1658/1659	c6744	321	y	638	chr-2	1.00E-90	8,178,536	chr-2	2.00E-118	7,332,649	no
1704/1705	c28392	230	n	not found	No hit			chr-2	9.00E-30	8,412,330	no
1573/1574	c1472	456	y	613	chr-2	6.00E-41	9,308,984	chr-2	4.00E-139	8,573,318	N/A
1642/1643*	c2838	199	n	1116	chr-2	2.00E-77	11,446,435	chr-9** BGI chr-2	2.00E-121	44,511,972 ~10,400,000	no
1692/1693* (h)	c19109	235	n	657	chr-2	3.00E-78	20,718,640	chr-2	2.00E-88	15,545,094	no
1510/1511*	c583	408	y	967	chr-2	7.00E-114	21,622,996	chr-2	0	15,729,820	no
					chr-2	Centromere region*	~30,000,000–35,000,000	chr-2	Centromere region*	~17,000,000–20,000,000	
1542/1543* (h)	c1312	332	y	2430	chr-10	5.00E-171	1,611,963	chr-2	0	21,705,415	no
1664/1665* (h)	c9776	333	n	6066	chr-7	0.00E+00	18,750,193	chr-2	0	22,231,687	no
1530/1531* (h)	c1279^b^	535	y	1669	chr-2	1.00E-85	45,159,033	chr-2	3.00E-140	24,209,378	no
1666/1667	c14318	366	y	1799	chr-2	1.00E-83	49,436,750	chr-2	0	24,923,646	N/A
1534/1535*(h)	c2785	313	n	887	chr-2	5.00E-33	52,122,450	chr-2	4.00E-104	26,193,530	N/A
1492/1493*	c2448	367	n	595	chr-2	4.00E-102	59,163,077	chr-2	0	30,619,621	no
1571/1572*	c6131	377	n	1406	chr-2	0.00E+00	62,313,486	chr-2	0	34,471,990	yes
1480/1481*	c13157	249	n	1392	chr-2	4.00E-30	62,939,050	chr-2	6.00E-111	34,862,608	yes
1512/1513* (h)	c8165	200	n	2167	chr-2	4.00E-128	63,654,052	chr-2	0	35,734,036	yes
1502/1713*	c3993	723	y	smaller	chr-6	1.00E-91	55,228,864	chr-2	3.00E-102	36,457,704	yes
1724/1725	c33813	229	n	692	chr-2	1.00E-81	64,442,929	chr-2	5.00E-70	36,736,857	N/S
1640/1641	c2807	331	n	smaller	No hit			chr-2	3.00E-125	37,436,112	N/S
1486/1487	c13922	360	n	571	chr-2	1.00E-63	65,545,284	chr-2	3.00E-128	37,909,036	yes
1630/1631*	c10535	243	n	3144	chr-2	6.00E-115	65,959,535	chr-2	6.00E-172	40,711,360	yes
1482/1483*	c13655	242	n	1592	chr-7	5.00E-156	59,786,300	chr-2	0	38,785,839	yes
1656/1657	c6373	257	y	not found	chr-2	1.00E-28	67,249,943	chr-2	4.00E-100	39,733,092	yes
1650/1715	c4150	497	y	1245	chr-2 chr-10	0.00E+00 0.00E+00	67734730 8795407	chr-2	0.00E+00	40,261,848	yes
1532/1533*	c7587	460	y	2203	chr-2	0.00E+00	68,388,845	chr-2	0.00E+00	40,983,241	yes
1581/1582	c32589	240	n	921	chr-2	4.00E-41	69,077,003	chr-2	4.00E-136	41,654,883	yes
1505/1716	c4364	223	n	569	chr-2	3.00E-122	69,975,526	chr-2	0.00E+00	42,480,974	N/S
1548/1549* (m)	c338	518	y	754	chr-2	0	70,344,223	chr-2	0	42,859,260	N/S
1690/1691	c1878	287	n	1583	chr-2	4.00E-160	70,622,801	chr-2	0	43,138,453	yes
1575/1576* (m)	c2388	207	n	1118	chr-2	0.00E+00	71,136,317	chr-2	0	43,636,319	yes
1506/1507*	c5080	383	y	1136	chr-2	3.00E-144	72,066,759	chr-2	4.00E-169	44,450,743	yes
1500/1501*	c3546	309	n	1538	chr-2	1.00E-125	72,255,129	chr-2	3.00E-140	44,616,393	yes
1646/1647	c3609	406	y	465	chr-2	7.00E-26	72,610,707	chr-2	3.00E-103	44,941,727	yes
1652/1653*	c5210	398	y	940	chr-2	0.00E+00	73,140,677	chr-2	0	45,462,728	yes
1670/1671	c2552	614	y	not found	chr-2	7.00E-95	73,707,837	chr-2	0	45,964,151	yes
1583/1681*	c1406	505	y	1405	chr-2	0.00E+00	73,764,842	chr-2	0	46,006,364	yes
1496/1497	c30198	225	y	2061	chr-2 chr-3	0.00E+00 0.00E+00	74,461,686 10,629,910	chr-2 chr-5	0.00E+00 0.00E+00	46,598,403 5,131,021	N/A
1484/1485	c1372	441	y	1843	chr-2	0.00E+00	75,033,568	chr-2	0	47,026,636	yes
1540/1541* (m)	c3455	327	n	4130	chr-2	0	75,576,016	chr-2	0	47,430,665	yes
1654/1655* (m)	c5851	228	n	557	chr-2 chr-1	2E-36 2E-29	75871067 58887780	chr-2	2.00E-55	47,643,065	yes
1708/1709	c704	675	y	1830	chr-2	0.00E+00	77,649,034	chr-2	0	49,009,115	N/S
1528/1529	c2339	383	n	3652	chr-2	0.00E+00	77,783,329	chr-2	0	49,087,867	No
1696/1697	c22381	185	n	1265	chr-2	1.00E-63	77,862,634	chr-2	3.00E-53	49,107,338	No

^a^Primer information from [13].

*BAC clones were isolated for these markers. (m) and (h) denote BAC clones with medium and high repetitive DNA. Underlined PS26 contig BAC clones were used for physical mapping.

^b^BAC clone did not give a single FISH signal. N/A–marker not scored. N/S–marker not specific in 312 segregating line.

Southern blot hybridization of *Hind*III digested fragments of BAC clones with ^32^P label genomic DNA from apomictic BC_8_ (06-A-58) was used to assess the level of DNA repetitiveness within the BAC clones based on signal intensity. A centromeric BAC clone was identified from the polyhaploid BAC library using a 160 bp *Kpn*I repeat probe [17].

### FISH

#### FISH probes

BAC DNA for nick translation was extracted using an alkaline lysis method http://www.protocolpedia.com/component/sobipro/?pid=69&sid=2209:BAC-DNA-Isolation-from-200-ml-Cultures-by-a-Cleared-Lysate-Method-Followed-by-Double-Acetate-Precipitation&Itemid=0 with the following modifications. The RNase treatment was done with 10 μl Ambion® RNase cocktail (Life Technologies, Grand Island, NY) consisting of 5 U of RNase A and 200 U of RNase T1. The BAC DNA was suspended in a final volume of 50 μl Buffer EB (QIAGEN Inc., Valencia, CA, USA). 1–2 μg of BAC DNA was labeled with biotin (bio)-11-dUTP (Roche, Indianapolis, IN) or digoxigenin (dig)-11-dUTP (Roche), using the nick translation kit (Roche) according to manufacturer’s instructions.

PCR centromere probes were prepared by labeling with biotin-11-dUTP using primers 5’-GGTACCCCGTAATAGTGCATTC-3’ and 5’-GAAAATGGTTTCGCAACAAAAG-3’ designed from the 160 bp *Kpn*I repeat family sequence [17].

#### Chromosome preparation

Root tips from apomictic BC_8_ (06-A-58) derived seedlings were collected, washed, placed in a 0.5ml Eppendorf tube with a hole in the lid in 300 μl distilled water and treated with nitrous oxide at 1 to 1.5 Mpa for 3 to 4 hours at room temperature in a Nitrous Oxide gas chamber [18] prior to fixing in 3:1 (V:V) ethanol to acetic acid solution. Root caps were removed and 2–3 mm of the meristematic region was incubated in an enzyme mix containing 2% (w/v) cellulose RS (Karlan Research, Santa Rosa, CA), 1% (w/v), pectolyase Y23 (Karlan Research, Santa Rosa, CA), 1% (w/v) macerozyme R 10 (Desert Biologicals, Phoenix, AZ) in citrate buffer (10 mM sodium citrate, 10 mM sodium EDTA, pH 5.5) [19] for 90 minutes at 37°C. Slide preparation for chromosome spreads after digestion was done either through air-drying [20] or a “SteamDrop” method [21].

#### Fluorescence *in situ* hybridization and detection

FISH was performed according to Zhong [22] with modifications. Slides with chromosomal spreads were treated with 5 μg/ml pepsin in 0.01M HCl for 5 to 10 minutes, fixed in 1% formaldehyde with 50 mM MgCl_2_ in 2x SSC and dehydrated in a series of 70, 90 and 100% (v/v) ethanol. The hybridization mix consisted of 1–5 ng/μl of each probe, 50% formamide, 10% dextran sulfate, 75–85 ng/μl salmon sperm DNA and 2× SSC in a final volume of 18–20 μl. If necessary, *P*. *squamulatum* blocking DNA (10–50 ng/μl) was added to the hybridization mix to block signal from minor repetitive sequence within BAC clones. Hybridization mixtures were denatured at 80°C for 5 minutes, snap chilled on ice, applied to the chromosome spread, and covered with a 22 × 30 mm coverslip. Slides were placed on an 80°C heat block for 3 minutes then incubated in a moist chamber at 60°C for 90 minutes followed by a 37°C incubation for 64–67 hours. Two post-hybridization washes were done in 50% formamide in 2× SSC at 37°C for 10 minutes each. Slides were blocked in TNB (100 mM Tris-HCl, pH 7.5; 150 mM NaCl; 1× DIG blocking solution, Roche) for 30 minutes at 37°C, and blocked again in 5% (w/v) IgG-free bovine serum albumin (Sigma) in TN (100 mM Tris-HCl, pH 7.5; 150 mM NaCl) for 30 minutes at 37°C. Two-color detection was carried out according to Zhong *et al*. (1996) with modifications. The biotin-labeled probes were detected with Texas red using a three step amplification and DIG-labeled probes were detected with FITC with a two-step amplification. Antibodies were diluted in TNB. Preparations were counterstained by mounting in Vectashield (Vector Laboratories) containing 1.5 μg/ml DAPI. Slides were examined with a Zeiss Axioskop 2 *plus* fluorescence microscope. Fluorescent signals were detected for DAPI (λ_ex_ = 360 nm, λ_em_ = 420 nm), FITC (λ_ex_ = 480 nm, λ_em_ = 515 nm), and Texas red (λ_ex_ = 560 nm, λ_em_ = 645 nm). Monochrome digital images were captured using a charge-coupled device AxioCam camera and stored using AxioVision, version 4.8 for Windows. Composite images were constructed using Adobe® Photoshop CS2 version 9.0.

### Deletion Study

#### Pollen irradiation

Pollen was collected in glassine bags from individual heads each morning between 10:30 and 11:00 am from greenhouse-grown plants. Individual plants were derived from six backcross 8 lines and one backcross 7 line, genotyped as ASGR positive. Pollen was irradiated within 30 minutes of collection in the glassine bags using a J.L. Shepherd Model 109-GR-12 self-contained Cobalt-60 irradiator set for either 2 or 3 Kr of Cobalt-60 gamma radiation. After irradiation, the pollen was immediately used to pollinate inflorescences of sexual tetraploid pearl millet which had stigmas exserted, but at least a day before pollen shed.

#### Molecular screen of plant lines derived from irradiated pollen crosses

DNA was extracted from equal amounts of tissue from 4 green plants within a line using a modified CTAB method [14]. DNA was then amplified with ASGR specific primers 787/788 and ASGR-carrier chromosome CAPS marker p1670/71 and SCAR marker 1656/1657 [13].

## Results and Discussion

Longer sequences were identified for 44 of the 49 ASGR-carrier chromosome 454 contigs within the Velvet assemblies (Table 1, S1 File). The Velvet-assembled contigs had to share at least 90% or greater sequence identity to the ASGR-carrier chromosome 454 contigs [13]. The longest available ASGR-carrier chromosome contig was used for BlastN *(*cutoff of *e*^-20^) *in silico* analysis against the foxtail millet (NW_004675962.1) and sorghum (NC_012877.1) genomes at the National Center for Biotechnology Information (NCBI). With the additional contig lengths, 47 and 44 of the ASGR-carrier chromosome contigs showed similarity to the foxtail millet and sorghum genomes, respectively. Forty-three (88%) and 37 (82%) of the ASGR-carrier chromosome contigs showed a unique or highest similarity to foxtail millet and sorghum chromosome 2 with individual contig hits distributed along the length of chromosome 2 of both species (Table 1). Contig PS26_c9369, tightly linked to the ASGR [13], had similarity to chromosome 1 in foxtail millet and chromosomes 3, 4, 6, and 10 in sorghum. PS_c194, PS_c28392 and PS_c2807 had similarity to foxtail millet chromosome 2 but did not have corresponding BlastN hits to the sorghum genome. PS_c9776, PS_c9993 and PS_c13655 had hits to foxtail millet chromosome 2 but identified more significant similarity to genes on sorghum chromosomes 6 or 7. These noted PS contigs did not tightly cluster in a particular area of the foxtail millet chromosome. PS26_c283, aligned on sorghum chromosome 2 but to foxtail millet chromosome 9, although it was identified on a scaffold mapped to chromosome 2 in the Beijing Genomics Institute (BGI) foxtail millet genome assembly (http://foxtailmillet.genomics.org.cn). The identification of large-scale collinearity between sorghum and foxtail millet for chromosome 2 was expected based on whole-genome dot plot comparisons which show that chromosome 2 in sorghum and foxtail millet share large degrees of similarity except at the centromeric region [4]. While the pearl millet genome is not yet available, comparative mapping revealed that pearl millet linkage group 7 is homoeolgous to foxtail millet 2 [23] and is likely the homoeolgous chromosome for the *P*. *squamulatum* ASGR-carrier chromosome.

**Fig 1:**
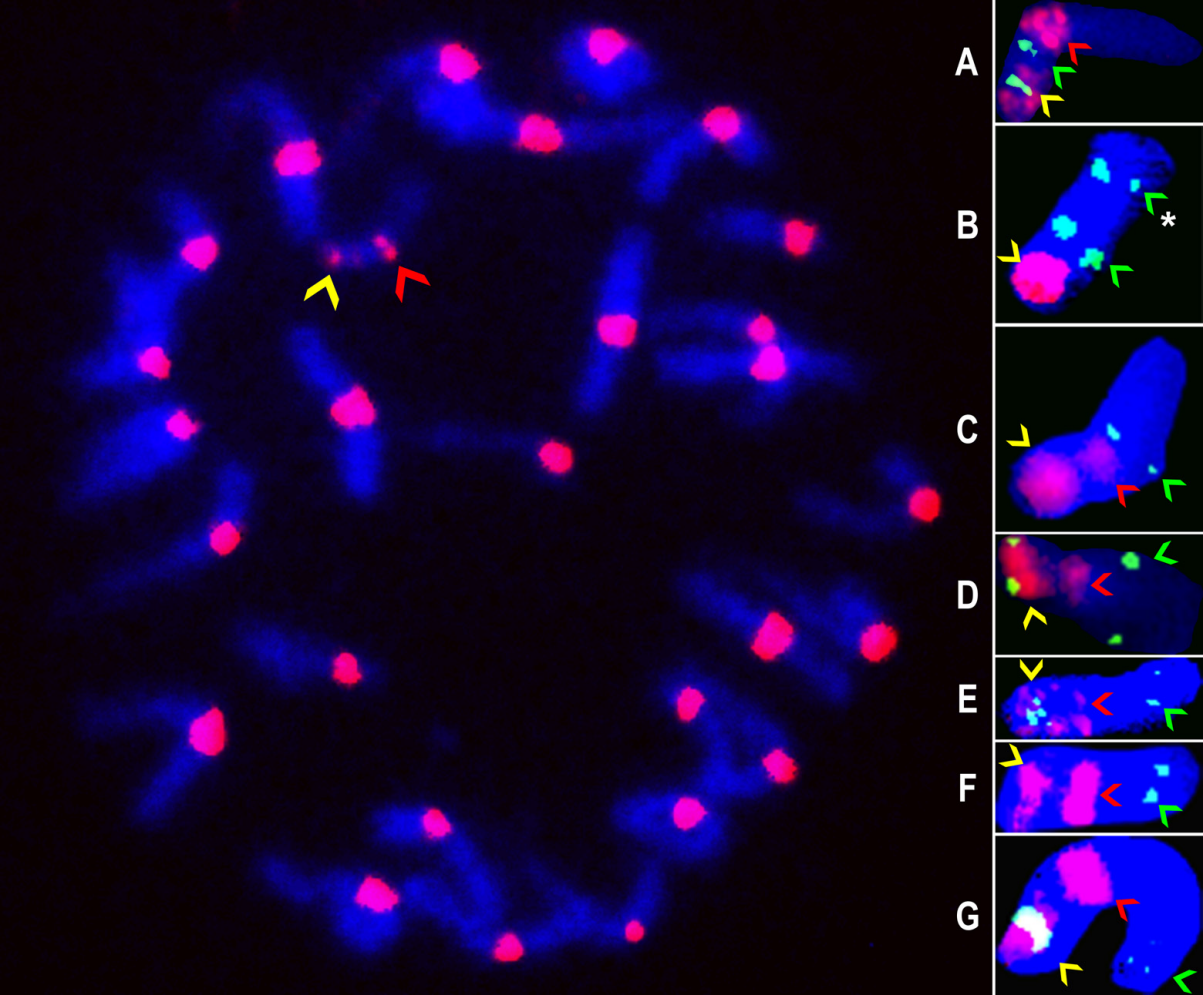
FISH images of BAC probes mapped to the ASGR-carrier chromosome. Example of a mitotic chromosome spread of apomictic BC_8_ (06-A-58) with the ASGR identified with a yellow arrow and the centromere of the ASGR-carrier chromosome identified by a red arrow. A-G insets are mapped ASGR-carrier chromosome BACs. The yellow arrow denotes the ASGR signal. The ASGR signal was identified either through a high copy ASGR-BAC clone (red pseudo-color) or a combination of a high (red pseudo-color) and low (green pseudo-color) ASGR-BAC clone. The red arrow denotes the centromere signal and the green arrow denotes the mapped ASGR carrier chromosome BAC signal from the following a) p285J18/PS_c2838, b) p220A02/PS_c583 and p236E19/PS_c10535 (*), c) p036L06/PS_c2448, d) p258L05/PS_c3993, e) p236E19/PS_c10535, f) p057M05/PS_c5080, and g) p142D19/PS_c5851.

A range of one to five BAC clones were isolated from the polyhaploid BAC library [15] for 25 of the 49 ASGR-carrier chromosome transcripts (Table 1). The relative amount of repetitive DNA within each BAC clone was assayed by the signal strength and number of restriction fragments of the BAC DNA hybridizing to labeled 06-A-58 total DNA when compared to ASGR BAC clones p109 and p800 which were used as low and high copy controls, respectively [8]. BAC clones with moderately (m) or highly (h) repetitive DNA are noted in Table 1. The ASGR-carrier chromosome BAC clones were selected for physical mapping based on the *in silico* mapping of the contig to the foxtail millet and sorghum genomes and their level of repetitive DNA. Seven BAC clones were physically mapped to the ASGR-carrier chromosome in apomictic 06-A-58. The ASGR on the ASGR-carrier chromosome was detected using either a high copy ASGR-BAC (red pseudo color signal denoted by the yellow ASGR arrow) which hybridizes to the high copy regions of the ASGR [8] (Fig 1B, 1C and 1F) or a combination of a high and low copy ASGR-BAC (green pseudo color signal denoted by the yellow ASGR arrow Fig 1A, 1D, 1E and 1G) near the telomere of the ASGR-carrier chromosome. A centromere probe was used in most FISH experiments (red pseudo color signal denoted by the red arrow). The position of the ASGR-carrier chromosome BACs are green pseudo color and denoted by a green arrow (Fig 1A–1G). Two of the seven ASGR-carrier chromosome BAC clones (Ps26_c2838 and Ps26_c583) mapped to the short arm of the ASGR-carrier chromosome while the remaining BAC clones mapped to the long arm. This result agrees with the *in silico* prediction based on centromere positions on chromosome 2 of sorghum and foxtail millet at ~30–35 Mb and ~17–20 Mb, respectively (http://ensembl.gramene.org). Using dual labeling of BAC probes, the order of PS_c3993/PS_c10535, PS26_5080 and PS26_c5851 was verified as linear from the centromere to telomere on the long arm of the ASGR-carrier chromosome. The signal order of PS_c3993 and PS_c10535 to each other was not verified. Our attempt to physically map the PS26_c1279 BAC, which *in silico* maps closer to the centromere on the long arm of the chromosome, was unsuccessful, even with blocking, due to repetitive DNA, which hybridized as a large signal on both sides of the centromere on the ASGR carrier chromosome.

**Fig 2:**
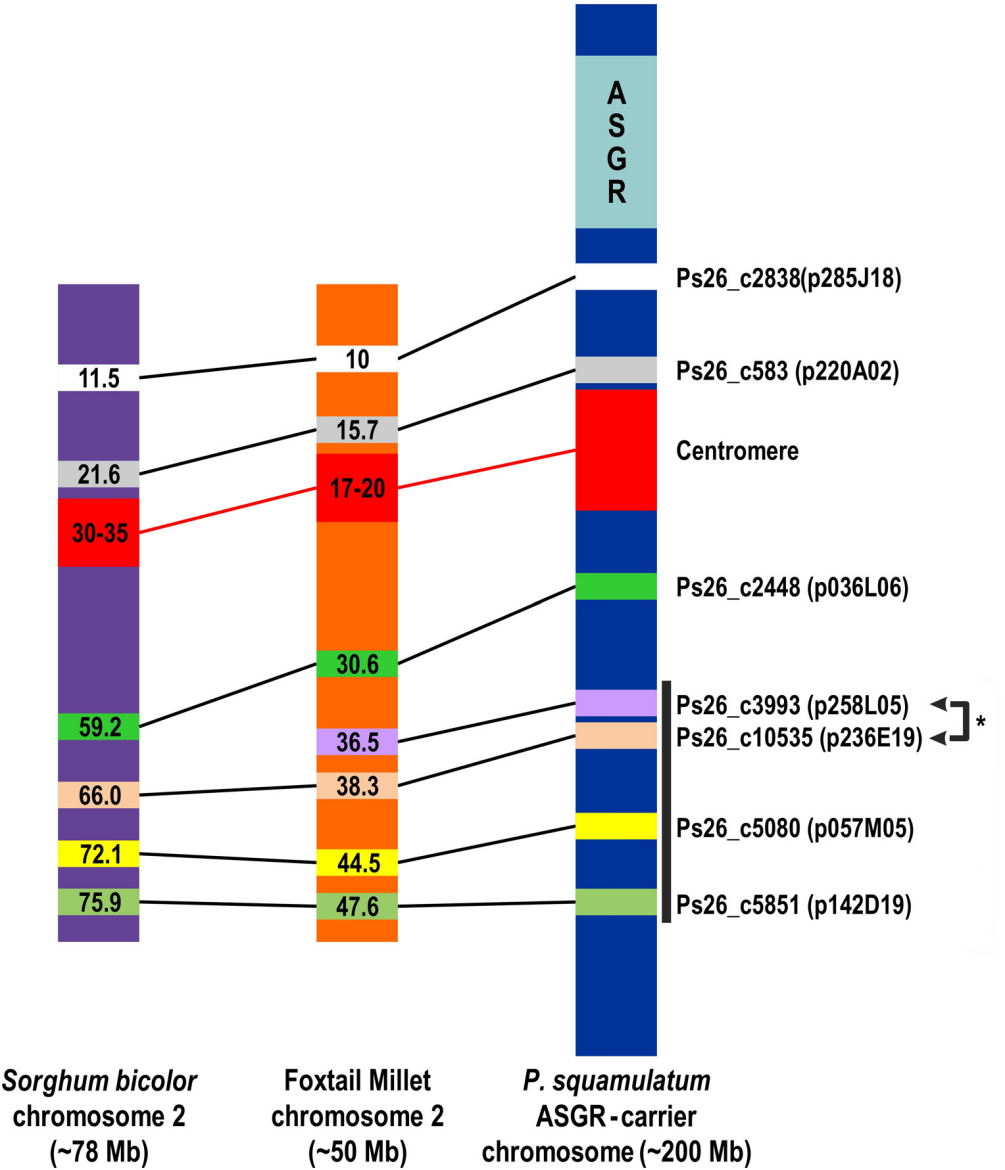
Diagram of collinearity identified using FISH signals on the *P*. *squamulatum* ASGR-carrier chromosome and the *in silico* positions in sorghum and foxtail millet chromosome 2. Chromosome lengths are not drawn to scale. Black box denotes BAC clones where linear order was confirmed. *Order of Ps26_c3993 and Ps26_c10535 BACs to each other was not verified.

Positions determined with cytogenetic mapping along the ASGR-carrier chromosome corresponded to the predicted *in silico* locations based on the sorghum/foxtail millet genomes as shown in Figs 1 and 2. Based on the *in silico* and cytogenetic mapping data, the collinearity of the *P*. *squamulatum* ASGR-carrier chromosome outside the ASGR and foxtail millet starts from ~5.5 Mb of foxtail millet and continues across the entire chromosome. Whether the synteny extends to the very beginning of chromosome 2 to right outside the ASGR remains unknown until additional BACs can be identified. Of the seven *P*. *squamulatum* ASGR-carrier chromosome BACs used for physical mapping, none showed additional FISH signals on the pearl millet chromosomes. The lack of signal on the pearl millet genome could be due to FISH hybridization conditions or to the divergence of the non-genic regions between the *P*. *glaucum* and *P*. *squamulatum* chromosomes. Additional research will be required to determine if the ASGR-carrier chromosome markers will be useful to identify similar regions in other apomictic *Pennisetum* species. If the ASGR-carrier chromosome BACs show signal on other *Pennisetum* species, this may allow us to more fully understand the evolution of the apomixis locus within the *Pennisetum* species.

A published RFLP mapping study placed the apospory locus on linkage group 7b in buffelgrass (*C*. *ciliaris*) [24]. Sequence information could be found for 3 RFLP markers within the 6 marker linkage group covering 78.8 cM. Two markers, HHU27 (gb|H54993.1) and pPAP3A07 (gb|BM084123.1) flanked the aposporous locus at 10.7 cM and 1.4 cM, respectively. The third marker, pPAP08H05 (gb|BM084577.1) resided 43.2 cM from HHU27. All 3 markers showed highest similarity in a linear order to chromosome 7 in foxtail millet and chromosome 6 in sorghum. In foxtail millet, pPAP3A07 was located at ~14 Mb, HHU27 at ~24 Mb and pPAP08H05 at ~31 Mb. In sorghum, pPAP3A07 was located at ~19.1 Mb, HHU27 at ~50.8 Mb and pPAP08H05 at ~58.4 Mb. While limited in data points, the marker sequence comparison and karyotype differences identified between the ASGR-carrier chromosomes in *C*. *ciliaris* and *P*. *squamulatum* [9, 10] further supports the idea of translocation of the ASGR to different chromosomes between apomictic *Pennisetum/Cenchrus* species. Changes at the apomixis-controlling-locus (ACL) in the *Paspalum* genus have also been identified. Markers from the telomeric portion of the long arm of rice chromosome 12 flank the ACL in mapping studies with *P*.*simplex* [25] and *P*. *malacophyllum* [26]. However, *P*. *notatum* showed both rice chromosome 2 and chromosome 12 markers flanking the ACL [26, 27]. Markers identified as ACL linked in *P*. *simplex* were not linked to the ACL in *P*. *procurrens* [28]. Sorghum chromosome 2 shows synteny blocks with rice chromosome 3, 7, 8 and 9, but not with rice chromosome 2 or 12 [29].

The DNA content of the *P*. *squamulatum* chromosome was roughly estimated at ~200 Mbp [8] of which ~50 Mbp is the ASGR. Therefore the *P*. *squamulatum* ASGR-carrier chromosome has expanded roughly 2 to 3 times when compared to the corresponding sorghum and foxtail millet chromosome 2. It is likely that much of that expansion is caused by transposable elements as has been shown when comparing the number of predicted genes within a genome to genome size in many plant species [30].

With the location of the ASGR-carrier chromosome markers identified, a screen to identify backcross lines with potential structural changes to the ASGR-carrier chromosome was undertaken. We sought to identify a line where a functional ASGR locus had been moved from the *P*. *squamulatum* ASGR-carrier chromosome to a pearl millet chromosome via irradiated pollen (S1 Fig). For the study, irradiated pollen from offspring derived from six different apomictic backcross 8 and one apomictic backcross 7 tetraploid pearl millet lines was used to pollinate 71 sexual tetraploid IA4X heads. As the ASGR is a single-dose locus, approximately half of the pollen used in the crosses would not carry the ASGR. To help identify lines which reproduced sexually, heads from 1962 plants derived from the irradiated pollen by IA4X cross were pollinated with Red IA4X pollen and seed collected. Red IA4X pollen contains the dominant *Rp1* allele which confers a red midrib color [31]. Plants producing only red progeny, indicating obligate sexuality, were not screened by DNA markers. Seventy-eight lines were initially tested for structural changes to the ASGR-carrier chromosome using ASGR SCAR marker p787/788 and ASGR-carrier chromosome SCAR markers from PS_c6373 (*in silico* mapped approximately half way between the centromere and telomere on the long arm of foxtail millet and sorghum) and CAPS marker from PS26_c2552 (*in silico* mapped close to the telomere of the long arm in foxtail millet and sorghum) based on their production of green, and therefore potentially apomictic, progeny. The markers chosen for screening are co-dominant and therefore would eliminate false negative results in the PCR- based screen. Fifty-one lines did not carry either the ASGR or ASGR-carrier chromosome markers tested. These lines producing green progeny were generated by self-pollination after unsuccessful crossing with Red IA4X pollen. Twenty-six lines carried all three markers and therefore did not contain large structural changes to the ASGR-carrier chromosome. One line, 312, contained both ASGR-carrier chromosome markers but not the ASGR marker. Line 312 was derived from 2 Kr irradiated pollen from the BC_7_ line. Plant 312 was screened for reproductive phenotype by ovary clearing [32]. As expected, plant 312 formed mature sexual embryo sacs. Further screening using the ASGR-carrier chromosome SCAR markers showed that the 312 plant inherited most of the long arm of the ASGR-carrier chromosome from *P*. *squamulatum* (Table 1).

As shown in our preliminary screen of gamma irradiated apomictic pollen offspring, the mapped ASGR-carrier chromosome markers can be used to identify structural changes in the ASGR-carrier chromosome as found in plant 312. Additional screening of more plants may allow us to identify apomictic lines with large deletions in the *P*. *squamulatum* ASGR-carrier chromosome. If found, these plants could be subjected to both genomic and transcriptional sequencing which could help as a process of elimination to identify the genes controlling apomixis in *P*. *squamulatum*.

## Supporting Information

S1 Fig Graphical Overview of Deletion Study Screen. Graphical overview detailing the steps of the deletion study screen.
(TIF)

S1 File PS_Contigs.txt. Fasta file of PS_contigs.
(TXT)
