# Supplementary figures and images for: In Silico and Fluorescence In Situ Hybridization Mapping Reveals Collinearity between the Pennisetum squamulatum Apomixis Carrier-Chromosome and Chromosome 2 of Sorghum and Foxtail Millet
Source: PLoS One. 2016 Mar 31;11(3):e0152411. doi: 10.1371/journal.pone.0152411 (PMC4816547; doi:10.1371/journal.pone.0152411)

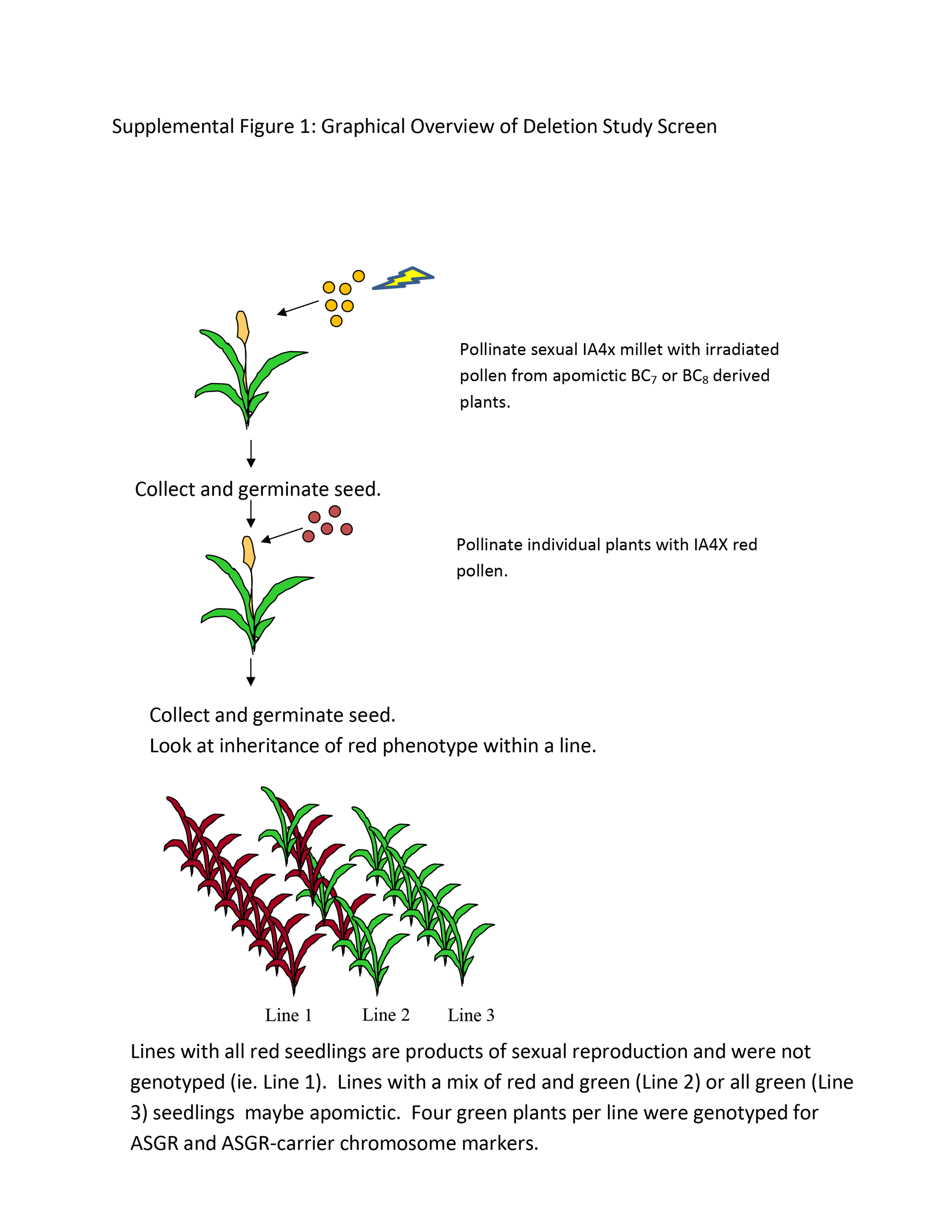

Supplement: S1 Fig — Graphical overview detailing the steps of the deletion study screen. (TIF) [file pone.0152411.s001.tif]
